# Immunoelectron Microscopic Evidence for Tetherin/BST2 as the Physical Bridge between HIV-1 Virions and the Plasma Membrane

**DOI:** 10.1371/journal.ppat.1000749

**Published:** 2010-02-05

**Authors:** Jason Hammonds, Jaang-Jiun Wang, Hong Yi, Paul Spearman

**Affiliations:** 1 Department of Pediatrics and Department of Microbiology and Immunology, Emory University, Atlanta, Georgia, United States of America, and Children's Healthcare of Atlanta, Atlanta, Georgia, United States of America; 2 Robert P. Apkarian Integrated Electron Microscopy Core Laboratory, Emory University, Atlanta, Georgia, United States of America; Northwestern University, United States of America

## Abstract

Tetherin/BST2 was identified in 2008 as the cellular factor responsible for restricting HIV-1 replication at a very late stage in the lifecycle. Tetherin acts to retain virion particles on the plasma membrane after budding has been completed. Infected cells that express large amounts of tetherin display large strings of HIV virions that remain attached to the plasma membrane. Vpu is an HIV-1 accessory protein that specifically counteracts the restriction to virus release contributed by tetherin. Tetherin is an unusual Type II transmembrane protein that contains a GPI anchor at its C-terminus and is found in lipid rafts. The leading model for the mechanism of action of tetherin is that it functions as a direct physical tether bridging virions and the plasma membrane. However, evidence that tetherin functions as a physical tether has thus far been indirect. Here we demonstrate by biochemical and immunoelectron microscopic methods that endogenous tetherin is present on the viral particle and forms a bridge between virion particles and the plasma membrane. Endogenous tetherin was found on HIV particles that were released by partial proteolytic digestion. Immunoelectron microscopy performed on HIV-infected T cells demonstrated that tetherin forms an apparent physical link between virions and connects patches of virions to the plasma membrane. Linear filamentous strands that were highly enriched in tetherin bridged the space between some virions. We conclude that tetherin is the physical tether linking HIV-1 virions and the plasma membrane. The presence of filaments with which multiple molecules of tetherin interact in connecting virion particles is strongly suggested by the morphologic evidence.

## Author Summary

Tetherin or BST2 is a cellular protein that was recently found to limit the ability of HIV to escape from cells. HIV counteracts this cellular restriction to its lifecycle by expressing the small viral accessory protein Vpu. Upon viral infection, cells expressing high levels of tetherin accumulate large clusters or strings of virions that remain attached to the plasma membrane by an unknown mechanism. The simplest explanation for this clustering is that tetherin itself physically attaches particles to the plasma membrane and to each other. In this article, we demonstrate that this is indeed the case. We found that particles released from cells by gentle protease treatment contain either cleaved or full-length tetherin. Using electron microscopy and immunogold staining, we show that tetherin is present as a physical link between viruses and the plasma membrane and sometimes between virus particles in large clusters or strings. Together this provides evidence that tetherin serves a direct, physical role in retaining particles on the surface of cells.

## Introduction

HIV interacts with a series of host proteins that facilitate its replication in cell. One of the clearest examples of this dependence on host machinery is the interaction between the p6 region of the HIV- 1 Gag protein and components of the cellular ESCRT machinery that are required for viral budding [1],[2]. Conversely, some host cell factors act to limit viral replication, and are collectively known as host restriction factors. Host cell restriction factors have been identified that target specific steps in the human immunodeficiency virus type 1 (HIV-1) lifecycle, including APOBEC3G [3],[4],[5], Trim5α [6],[7], and recently tetherin [8],[9]. These innate cellular defenses are constitutively expressed by host cells and can be upregulated in response to viral infection through the expression of type 1 interferons (IFNs). Viruses in turn have evolved to express adaptor molecules that counteract important host cell restrictions, as illustrated by the Vif protein of HIV, which enhances the proteasomal degradation of APOBEC3G, and the Vpu protein, which relieves the host restriction imposed by tetherin.

HIV-1 Vpu is a 16-kDa type 1 integral membrane protein [10],[11]. Vpu operates as a multifunctional adaptor protein causing surface down-regulation and proteasomal degradation of CD4 in infected T lymphocytes [12],[13],[14] and enhancing viral particle release [15],[16]. These two activities are separable, mapping to distinct structural domains and occurring in different subcellular compartments [17],[18]. The particle release activity of Vpu was noted long ago to be cell-type dependent [8],[19],[20],[21]. The presence of a host restriction factor acting at the level of particle release was suggested several years ago by experiments in which heterokaryons between restrictive and permissive cell lines exhibited a dominant restriction to particle release that was relieved by Vpu [16]. Recently the host cell restriction factor inhibiting particle release in the absence of Vpu was identified as bone marrow stromal cell antigen 2 (BST-2), also known as HM1.24, CD317, or tetherin [8].

Tetherin is a 28- to 36-kDa, type II integral membrane glycoprotein. The atypical topology of tetherin is comprised of a short N-terminal cytoplasmic tail, a single transmembrane spanning region, and a glycosyl-phosphatidlyinositol (GPI) anchor at its C-terminus. Tetherin's subcellular distribution is punctate on the plasma membrane and is also found on intracellular endosomal membranes, particularly the *trans*-Golgi network [22],[23]. Tetherin expression is constitutive in restrictive human cell lines, including HeLa, H9, Jurkat, Molt4, primary T lymphocytes, and primary macrophages, and is absent in cells that are permissive for particle release, such as 293T, HOS, and HT1080 [8]. Tetherin expression is IFN-inducible, and IFN treatment of permissive human cell lines results in a restrictive, Vpu-dependent particle release phenotype. Tetherin overexpression results in a dramatic accumulation of virion particles attached to the plasma membrane of cells infected with *vpu*-deficient viruses. Virions are often found clustered at focal areas on the plasma membrane, and appear to be attached in long linear arrays extending from these plasma membrane foci [8],[15]. As the name implies, tetherin is presumed to provide a physical tether between the plasma membrane and retained virions. However, the mechanism by which tetherin attaches nascent, mature HIV-1 virions to the plasma membrane remains to be fully elucidated. Tetherin and virions appear to colocalize by confocal microscopy at the particle budding site in some reports [8],[9],[24], but not in others [25]. Immunoelectron microscopy of HA-tetherin failed to demonstrate concentration of the protein at particle budding sites [25], and no definitive immunoelectron microscopic analysis of endogenous tetherin at the particle budding site has yet been reported. Furthermore, tetherin was recently found to be lacking on virions released from HeLa cells by shearing, suggesting that perhaps tetherin is not acting directly as a physical tether at all [26].

In this study, we provide evidence that tetherin is the physical linkage responsible for attachment of nascent HIV-1 virions to the plasma membrane. Partial protease stripping experiments utilizing both over-expressed and endogenous tetherin sources demonstrated incorporation into HIV-1 virions. In the absence of Vpu, tetherin was enriched on filamentous structures connecting virions to the plasma membrane, and was present between chains of tethered viral particles present in focal accumulations in infected cells. These results confirm tetherin's role in the physical attachment of virions to the plasma membrane and to each other in the absence of Vpu.

## Results

### Specific detection of endogenous tetherin in A3.01 cells following IFN treatment

**Figure 1:**
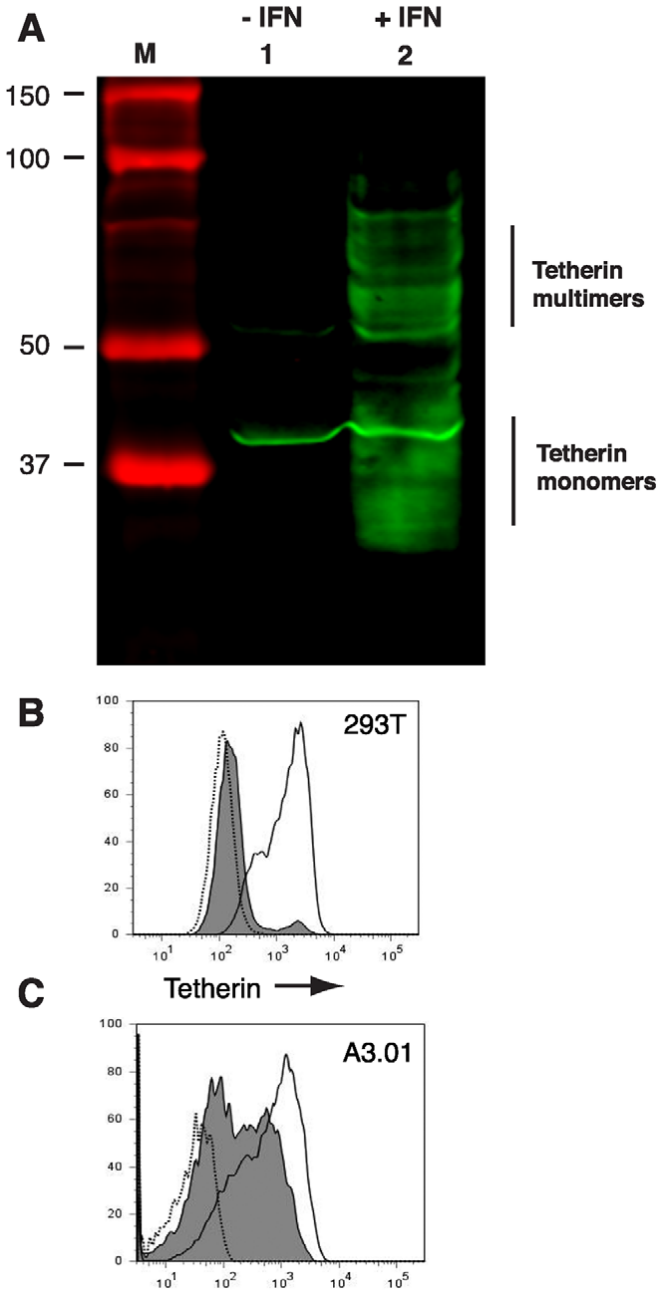
Interferon-α induces the expression of tetherin in A3.01 and 293T cells. (A) A3.01 cells were treated with 3000 U/ml of IFN-α for 24 hours (lane 2) and compared with untreated cells (lane 1). Cell lysates were harvested and analyzed by Western blotting using a rabbit anti-tetherin polyclonal antisera. Detection was performed via infrared detection on a LiCor Odyssey instrument. (B) 293T cells were treated overnight with 5000 U/ml of IFN-α (solid line) and the cell surface density of tetherin was then analyzed by flow cytometry. Unstimulated 293T cells (filled line) and a secondary antibody alone sample (dotted line) were analyzed as controls. (C) A3.01 cells were treated overnight with 3000 U/ml IFN- α. Solid unfilled line represents post-IFN cell surface labeling with anti-tetherin antiserum.

The human T cell line A3.01 was employed in the original description of the EM phenotype of T cells infected with *vpu*-deficient HIV-1 [15]. Because this original report showed clearly both the accumulation of tethered virions on the plasma membrane and the accumulation of virions in intracytoplasmic vacuoles consistent with the action of tetherin, we concentrated on this cell type for the experiments in this report. A3.01 cells were incubated for 24 hours in the presence of 3000 U/ml recombinant IFN-α to induce tetherin expression. A3.01 cell lysates expressed near undetectable levels of tetherin, while the IFN-α treated population exhibited substantial induction of tetherin expression as indicated by Western blot analysis (Fig. 1A). Tetherin was apparent as a series of bands at the 25–40 kD range, with a higher molecular mass component of apparent dimers. A background band at approximately 38 Kd is noted on this blot in both lanes. Miyagi and coworkers described a similar pattern for tetherin by Western blotting, but did not detect tetherin in A3.01 cells [26], perhaps attributable to differences in the polyclonal rabbit antisera produced and employed in our laboratory. We then wanted to further define the specificity of our antisera to detect endogenous tetherin under non-denaturing, non-reducing conditions. The permissive 293T cell line has been shown to express low levels of tetherin [8]. To induce tetherin expression, 293T cells were cultured in the presence of 5000 U/ml of recombinant IFN-α for 24 h and compared with control cells. Cell surface staining of tetherin and subsequent analysis by flow cytometry displayed a substantial induction of tetherin staining of the IFN-α treated population as compared to the untreated cell sample (Fig. 1B). A3.01 cells displayed two peaks when unstimulated, a tetherin-low and tetherin-intermediate population (Fig. 1C, grey peaks). Following IFN stimulation, a distinct shift to a more uniform population of tetherin-high cells was noted (solid line, no fill). IFN-α stimulated A3.01 cells demonstrated a restrictive phenotype that was consistent with the action of tetherin; this restriction was overcome by Vpu (Fig. S2). These data demonstrate the specificity of the rabbit anti-tetherin antiserum employed in this study, and indicate that IFN-α induction of tetherin in A3.01 cells induces a restriction to particle release that correlates with high cell surface levels of tetherin.

### Tetherin co-sediments with released HIV-1 particles

**Figure 2:**
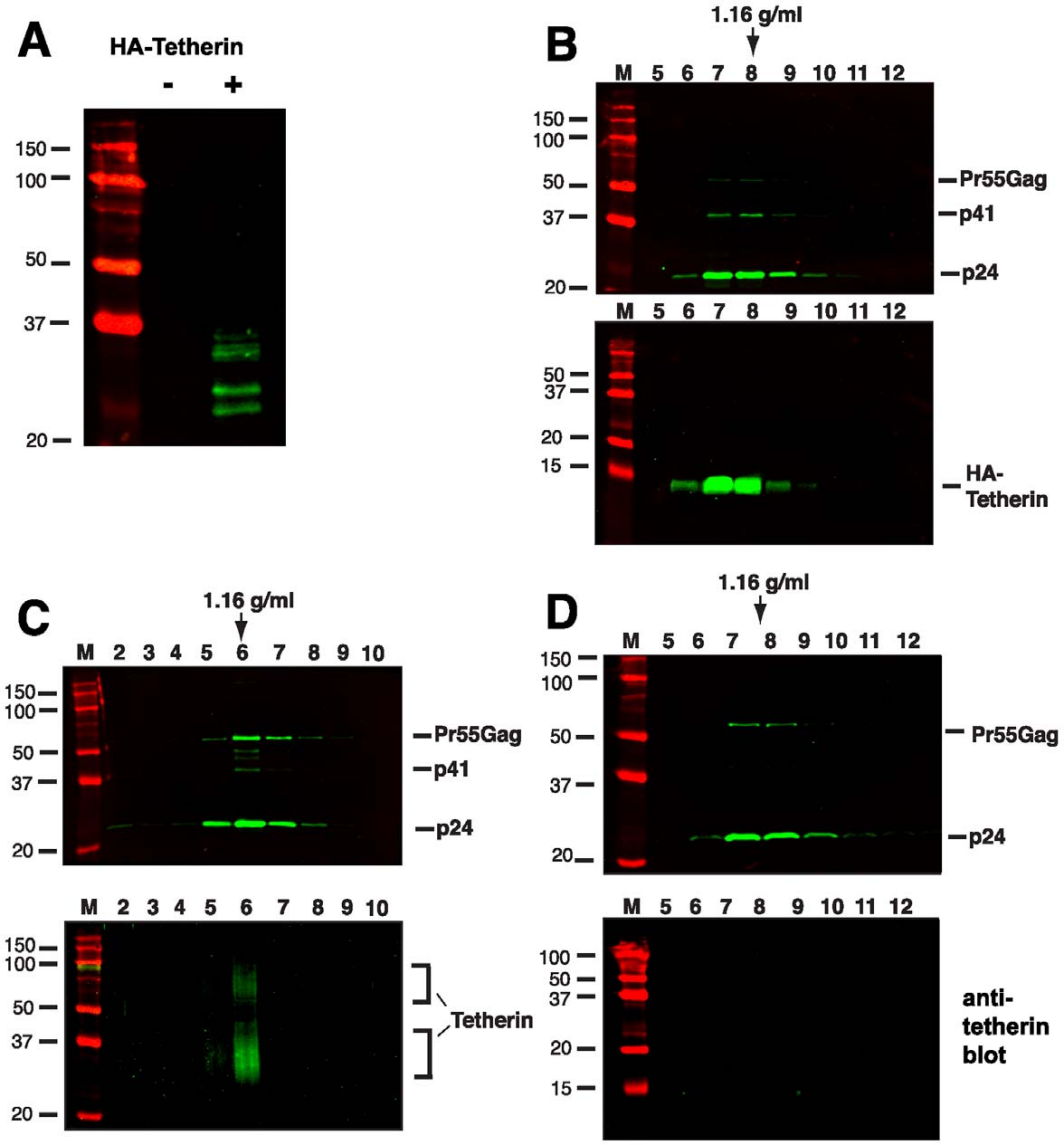
Tetherin is incorporated into virions released by protease treatment of NL4.3/Udel infected cells. 293T cells stably expressing an N-terminal, HA-tagged tetherin were transfected with NLUdel as described in Materials and Methods. (A) Cell lysate of 293T cells expressing HA-tetherin (+) or parental 293T cells (−), probed with anti-HA antibody. (B) At two days post-transfection, cell monolayers were digested with 5 ug/ml of subtilisin for one hour at 37°C. Supernatants were concentrated through a sucrose cushion and pelleted material was then layered onto a linear sucrose gradient. Fractions were analyzed by Western blotting using an anti-p24 monoclonal antibody (top blot) and anti-tetherin polyclonal antisera (bottom blot). (C) A3.01 cells were infected with VSV-G pseudotyped NLUdel and treated with 3000 U/ml of IFN-α at 3 days post-infection. The following day, IFN-stimulated cells were treated with 3 µg/ml TPCK-trypsin for 4 hours at 37°C. Supernatants were then analyzed as in (A). Density of peak gradient fraction is indicated by arrow. (D) NL4.3 virions released from IFN-stimulated A3.01 cells were harvested and analyzed by gradient centrifugation (without protease treatment).

The enrichment of tetherin on HIV-1 virions has not been established, and has not been detected by some investigators [26]. We thought it unlikely that tetherin would function as a physical link between particles and yet not be present in purified particle preparations. To begin to address the hypothesis that tetherin behaves as a physical linkage between nascent HIV-1 particles and the plasma membrane, we employed an assay to recover tethered virions from cell surfaces by proteolytic digestion [8]. We developed a 293T cell line stably expressing an N-terminal, HA-tagged tetherin (HA-tetherin). HA-tetherin cells were transfected with pNL4.3/Udel and cultured for an additional 48 hours. HA-Tetherin bands were apparent in 293T-tetherin cells at molecular masses ranging from 25–36 kD (Fig. 2A, + lane). Tethered virions were subtilisin “stripped” from the cell surface, and the supernatants concentrated through a 20% sucrose cushion. Concentrated material was then separated on linear 20–60% sucrose gradients by equilibrium density centrifugation. Stripped virions from this tetherin over-expression system were found to incorporate tetherin as evidenced by detection at a typical retroviral particle density enriched in Gag proteins (Fig. 2B). HA-tetherin on this blot formed a single dominant band at 13 Kd, representing a uniform cleavage product with a protected HA tag. This band was substantially smaller than the full-length HA-tetherin observed in untreated cell lysates (Fig. 2A). This is in fact the size that would be expected following cleavage at the predicted subtilisin cleavage site following residue 67 (motif RNVTH, residues 64–68 of tetherin). Recognizing that over-expression of a membrane protein could result in its incorporation into virions in a non-physiologic manner, we next sought to detect endogenous tetherin on released virions from infected T cells. A3.01 cells were infected with VSV-G-pseudotyped NL4.3/Udel, cultured for 48 h, incubated with IFN-α for an additional 24 hours, and then subjected to gentle proteolytic digestion using TPCK-treated trypsin. Protease stripped supernatants were again concentrated and separated on a linear sucrose gradient. Endogenous tetherin was enriched in the peak viral fraction and co-sedimented precisely with NL4.3/Udel virions (Fig. 2C). Interestingly, two major species of tetherin were detected on virions by Western blotting. The presence of both forms was consistent with Western blot analysis performed on IFN-α stimulated A3.01 cell lysates (Fig. 1A), and suggests that the gentle trypsin treatment released virions without cleaving all of the full-length, virion-associated tetherin. These data demonstrate that tetherin is incorporated onto virions, and would be consistent with the proposed role of tetherin in physically linking particles to the plasma membrane and to each other. We next examined wildtype particles for the presence of tetherin. IFN-stimulated A3.01 cells were infected with wildtype NL4.3, and particle gradients performed as for NL4.3/Udel. No tetherin was detected in the peak fractions from NL4.3 virions, indicating that they failed to incorporate endogenous tetherin due to the influence of Vpu (Fig. 2C). NL4.3-infected cells stripped with TPCK-treated trypsin released low amounts of virions and also failed to concentrate tetherin (data not shown). The incorporation of endogenous tetherin on NL4.3/Udel virions released by gentle protease digestion, and not wildtype NL4.3 virions, is consistent with a physical tethering of HIV virions that is overcome by Vpu.

### Tetherin is Concentrated along Filamentous Connections between HIV-1 Virions

**Figure 3:**
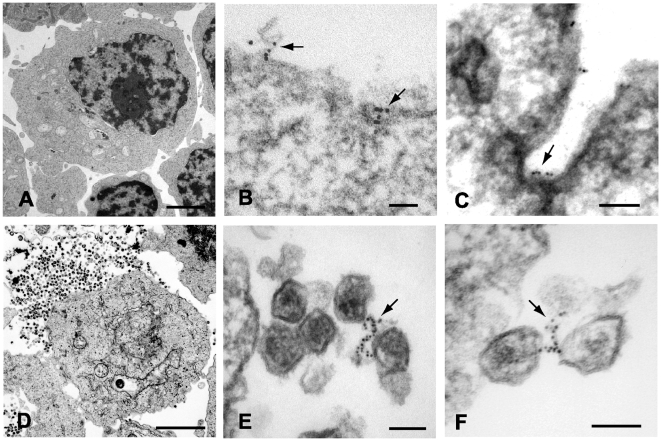
Immunoelectron micrographic analysis of tetherin on IFN-α stimulated A3.01 control cells and samples infected with pseudotyped NL4.3/Udel. (A) Uninfected control A3.01 cells, bar = 2.5 µm. (B) Six nanometer beads indicate focal tetherin immunostaining on the surface of IFN-α stimulated A3.01 cells. Labeling was performed after gentle fixation and prior to embedding. Arrows pointing to tetherin stained foci on plasma membrane extensions or pit. Bar = 50nm. (C) Focal tetherin staining within a membrane invagination or pit (bar = 100nm). (D) NL4.3/Udel-infected cells exhibit substantial accumulations of tethered mature HIV-1 virions (upper left, bar = 2 µm). (E and F) Six nanomolar beads indicate tetherin positive filaments connecting mature HIV-1 virions. Arrows exhibit significant tetherin staining on filamentous structures between mature viral particles tethered to plasma membrane. Bars = 100nm.

The results above are supportive of the idea that tetherin is present on particles and functions as a physical tether. However, we thought that more direct evidence would require demonstration of tetherin at the budding site and within virion-virion connections in the patches of tethered virions frequently seen when restrictive cells are infected with *vpu*-deficient HIV-1. We therefore performed immunoelectron microscopic analysis of tetherin in A3.01 cells under a variety of conditions, including unstimulated cells, cells that had been subjected to IFN-α stimulation, and following infection with NL4.3/Udel. Several techniques were pursued in order to achieve specific staining. We first performed immunostaining with polyclonal anti-tetherin antisera prior to embedding in cells that had been lightly fixed with paraformaldehyde. Cells were then extensively washed and exposed to secondary antibody conjugated to 6 nm gold particles, followed by fixation, embedding, sectioning, and examination by transmission electron microscopy. IFN stimulated A3.01 cells maintained typical T-lymphocytic morphology (Fig. 3A). Tetherin staining in uninfected cells was not diffuse, but was detected at focal membrane projections and small pits along the plasma membrane (Fig. 1B and 1C). Detection of tetherin by immunogold labeling in this manner in unstimulated cells was minimal (less than 0.1% of cells examined, Fig. S1A and S2A), while specific labeling was observed on 3.5% of IFN-stimulated cells (Fig. S2A). Infection of IFN-stimulated A3.01 cells with NL4.3/Udel resulted in vast focal accumulations of mature virions attached to the plasma membrane (Fig. 3D). Strikingly, within the tethered patches of virions, filamentous structures connecting mature virions exhibited positive tetherin immunogold labeling (Fig. 3E and F, arrows). In each case, the immunogold beads appeared to be arrayed upon a filamentous, electron-dense substrate. The apparent filaments were sometimes noted to link multiple virions together (Fig. 3F). We note that the length of the linearly-arrayed tetherin in these micrographs appears inconsistent with a simple protein bridge, but could represent tetherin on an extended membranous projection (see Discussion).

### Tetherin localizes to HIV-1 budding sites on the plasma membrane of infected cells

**Figure 4:**
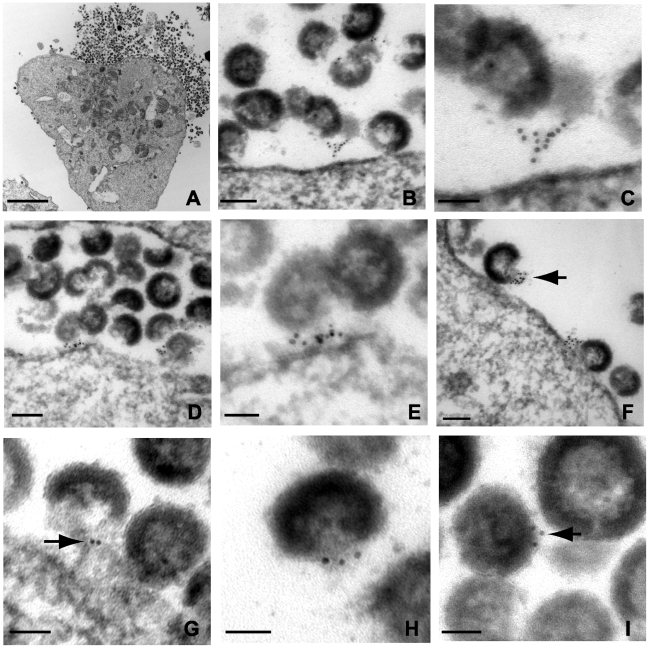
Immunoelectron micrographic analysis of tetherin of NL4.3/Udel-infected A3.01 cells treated with indinavir; labeling at site of particle budding. (A) Low magnification image of infected cells exhibiting accumulation of immature tethered virions, bar = 2µm. (B) Immunogold labeling of tetherin localized to particle budding sites on the surface of IFN-α stimulated cells. Bar = 100nm. (C) Higher power view from plasma membrane region exhibited in (B); bar = 50nm. Tetherin-positive filamentous structures are shown connecting immature particle to the plasma membrane. (D) Immunogold labeling of plasma membrane region exhibiting accumulation of tethered immature virions and significant labeling of membrane budding site. Bar = 100nm. (E) Higher power view of membrane proximal region from (D); bar = 50nm. HIV-1 virions attached to the plasma membrane exhibit significant tetherin immunolabeling. (F) Tetherin immunolabeling at virion budding locations along plasma membrane. Arrow indicates cluster of tetherin immunolabeling on particle membrane, at “open” end of virion shell. Bar = 100nm. (G) Higher magnification view of tetherin-positive staining at particle budding site. Bar = 50nm. (H) Additional view of tetherin immunolabeling on “open end” of immature virion. (I) Immunolabeling of tetherin associated with virion lipid envelope, bar = 50nm.

In order to generate robust tetherin immunogold labeling, A3.01 cells in the experiments above were only slightly fixed, causing aberrant virion morphology. In an attempt to improve virion morphology while addressing the same question, IFN-stimulated, NL4.3/Udel infected A3.01 cells were treated with indinavir to inhibit particle maturation, then stained prior to complete fixation and embedding as before. We reasoned that the stable and electron dense immature Gag shell would enhance this analysis. Indinavir treatment had no appreciable phenotypic affect related to the accumulation of virions along the plasma membrane in the absence of Vpu (Fig. 4A). Tetherin was concentrated at HIV-1 plasma membrane budding sites in infected A3.01 cells (Fig. 4B–G). Filamentous structures connecting particles immediately adjacent to the plasma membrane exhibited robust tetherin immunogold labeling were again observed (Fig. 4B and C). Where filamentous structures were not observed, tetherin was localized in discrete clusters at sites of active particle generation along the membrane of infected A3.01 cells (Fig. 4D–G). Again we noted that tetherin staining was not diffusely present along the plasma membrane. Also evident in these electron micrographs was a propensity of tetherin staining at the virion “base”, or areas of low particle electron density directed toward the pole of the virion adjacent to the plasma membrane (Fig. 4G and H). Tetherin staining was not restricted entirely to the particle budding site on the plasma membrane, however, and was readily observed on the virion envelope within clusters of virions that were not adjacent to the membrane in the observed section (Fig. 4I).

### Tetherin is the physical link between linear chains of HIV-1 particles

**Figure 5:**
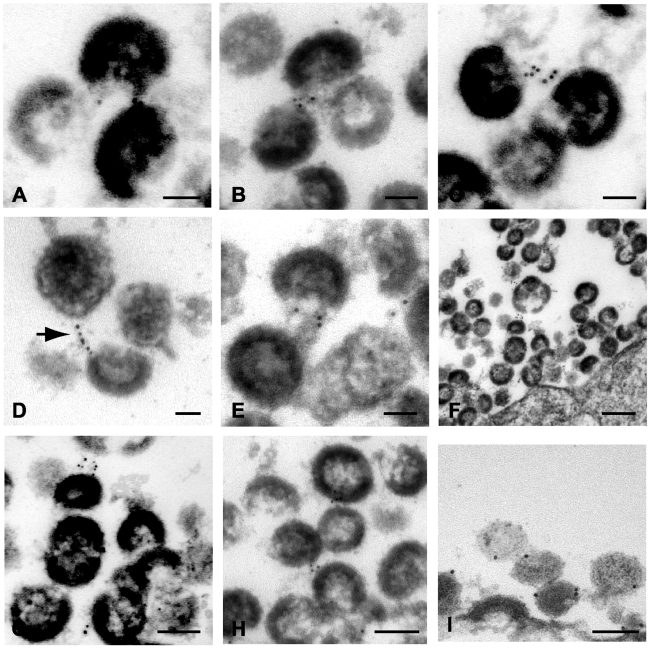
Immunoelectron micrographic analysis of indinavir-treated, NL4.3/Udel-infected A3.01 cells demonstrating inter-virion connections. (A–C) Tetherin immunolabeling between immature virions. Bar = 50nm. (D) Linear tetherin filament bridging virions. Bar = 50nm. (E) Immunolabeling of electron dense material between virions. Bar = 50nm. (F) Lower magnification image, demonstrating multiple points of tetherin labeling between particles in extended chains. Bar = 200nm. (G–H) Tetherin immunolabeling between chains of particles. (I) Alternative labeling of tetherin using the same rabbit antisera followed by 10nm protein A gold beads, demonstrating tetherin on outer surface of virion chains. Bars = 100nm.

We demonstrate above that tetherin is present in concentrated fashion at focal sites of particle budding. We next asked how the long strings of virions observed in many studies of *vpu*-deficient virions might form. Fig. 5 presents a series of micrographs in which immunogold staining was observed between virions within apparent chains or strings. Most common were the clustered immunogold beads between immature particles as shown in Fig. 5A and 5B. A second pattern was similar to the filamentous structures already described (Fig. 5C and 5D). The stacked, parallel appearance in some sections was suggestive to us of a helical filament cut in cross section (Fig. 5C), while in others a more linear extended connection was apparent (Fig. 5D). Tetherin staining appeared to be present within chains extending several microns away from the plasma membrane, and was not observed more than 50 nm away from particles (illustrated by Fig. 5F). To demonstrate that the presence of tetherin between virions was not due to aggregates or non-specific sticking of the anti-rabbit conjugated 6nm gold beads, we substituted 10nm protein A coated gold beads. Chains of NL4.3/Udel virions demonstrated 10nm gold beads connecting the virions to each other and to the plasma membrane, confirming the previous findings (Fig. 5I). In this experiment, extended filaments were not readily apparent, but rather the protein A gold was found directly between adjacent particles.

To further confirm the specificity of the observed labeling, identical experiments were performed in infected cells in the absence of labeling with the primary antibody. Strings of immunogold particles were not observed in over 50 sections examined from cells infected with NL4.3/Udel either with (Fig. S1D–F) or with indinavir (Fig. S1G). Individual gold particles were sometimes observed on the cell surface in these experiments, but at a much reduced frequency (detailed in Fig. S2A).

### Cryosectioning and immunogold labeling confirms direct tetherin association with particles

**Figure 6:**
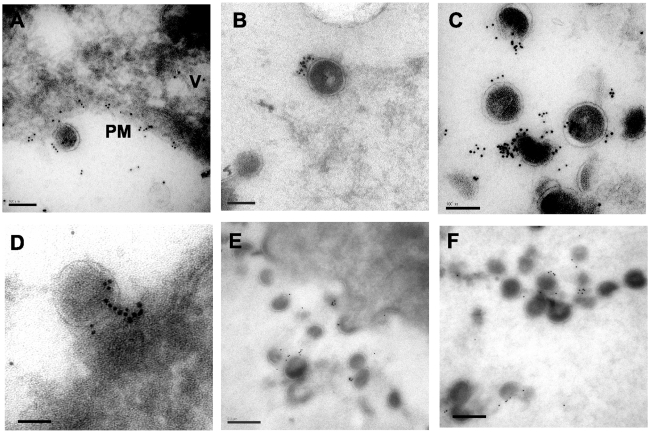
Immunoelectron micrographic analysis of cryosections from IFN-α stimulated, NL4.3/Udel-infected A3.01 cells. Sections were frozen in liquid nitrogen and sectioned by cryoultramicrotomy, then sections placed on nickel grids for immunolabeling with rabbit anti-tetherin antisera and a secondary antibody conjugated to 6 nm gold beads. (A) Focal section of plasma membrane (PM) and intracellular vesicle (V) demonstrate tetherin staining. Bar = 100nm. (B and C) Specificity of extracellular virion labeling with anti-tetherin labeling; bars = 100nm. (D) Concentrated tetherin staining at base of tethered particle on PM. Bar = 100nm. (E–F) Tetherin staining of cryosections following additional fixation step as described in Results. Intensity of staining is somewhat reduced, maintaining specificity for tethered chains of particles. Bar = 100nm.

A second technique to establish the relationship between tetherin and virion budding sites and particles was desirable. We continued this examination with A3.01 cells that had been infected with NL4.3/Udel and IFN-stimulated, but performed cryosectioning followed by immunolabeling with the identical polyclonal anti-tetherin antisera and secondary goat anti-rabbit antibody conjugated to gold. Staining was observed predominantly on the plasma membrane (PM, Fig. 6A) but also in apparent intracellular vesicles (V, Fig. 6A). A low level of apparent background staining was seen outside of cells (Fig. 6A). Importantly, the majority of virion particles observed were labeled with gold particles, and overall staining outside of 50 nm from particles was rare (Fig. 6B, 6C). We counted the number of gold beads associated with 100 consecutive virus particles labeled in the presence or absence of primary antibody. Background in the absence of primary antibody was exceedingly low (Fig. S1H and S1I), and the mean number of gold beads per virion stained with the anti-tetherin antibody was 9.3±5 (Fig. S2B), while the mean number of gold beads was 0.2±0.4 in control experiments omitting the primary antibody. Tetherin staining was specifically located at the base of viral particles bridging the virion membrane and plasma membrane, similar to what had been observed with the pre-embedding labeling technique (Fig. 6D). We slightly altered the fixation technique and repeated the cryosectioning and immunostaining experiment, (Fig. 6E and 6F). With this alteration in methods, very little background staining was observed, and 91% of gold beads were found to be located within 50nm of visible particles. The number of gold beads per virion in this experiment was 3.2±2.2, while using pre-immune sera the mean number of gold beads per virion (within 50nm) was 0.2±0.2 (Fig. S3, panels A–C). Taken together, these data are supportive of the role of tetherin as a physical link between the plasma membrane and virions, and suggest a potential role for a filamentous connection or substrate on which tetherin concentrates that contributes to the tethering phenomenon.

## Discussion

Tetherin is a restriction factor that restricts the release of retroviruses and filoviruses [27],[28],[29] by inducing their accumulation at the plasma membrane of infected cells. Vpu overcomes this restriction for HIV, while HIV-2 and SIVs have evolved distinct mechanisms to counteract the block. In the case of HIV-2 Rod10 isolate, the envelope glycoprotein counteracts tetherin, while Nef fulfills this function for a number of SIV strains [30],[31]. The mechanism by which Vpu lifts the restriction to particle release imposed by tetherin remains under study. Downregulation of cell surface tetherin was observed initially by the Guatelli laboratory [9], providing a conceptually simple model that remains under study. Downregulation of surface tetherin has not been universally observed despite the clear relief of restriction by Vpu [26]. Evidence for Vpu-mediated degradation of tetherin by proteasomal [25],[32] or lysosomal [24] pathways has been presented. The potency of the block to particle release induced by tetherin makes it an exciting area of investigation, both for its ability to enlighten understanding of intrinsic host restriction mechanisms and for its therapeutic implications.

The work presented here addressed a simple question: is tetherin the physical connection that links retained virions to the plasma membrane and to each other? Surprisingly, this had not yet been established, and some data to the contrary has been presented. During final revision of this manuscript, however, a paper strongly supporting tetherin as the physical link between virions and the plasma membrane was published [33]. In this paper, Perez-Caballero and coworkers demonstrate that tetherin is directly incorporated onto the membrane of HIV-1 particles. Our data agree with this finding, and support a model in which tetherin is present in the correct location to act as a physical tether. Tetherin is present at the budding site, it links particles together in long chains or strings, and it is present on released virion particles. These results certainly imply that removal of tetherin from the site of budding should remove the restriction, supporting the contention that cell surface downregulation of tetherin should correlate with relief of restriction. Results here were performed with endogenous tetherin in an infected T cell line, chosen because it is a prototypical cell line exhibiting a Vpu-responsive (tetherin-mediated) restriction to particle release. It will be important to repeat these experiments in infected primary T cells and macrophages, but we anticipate that the presence of tetherin at the site of particle retention will be similar.

In some of our experiments, an extended filamentous electron-dense structure formed a bridge between retained virions. These filamentous structures demonstrated strong immunolabeling with tetherin. The length and intensity of labeling of these filaments makes it clear that multiple tetherin molecules are present, and that they cannot be tetherin dimers alone. The distance covered by these linear structures is too great to represent a protein bridge formed by tetherin-tetherin interactions. It is possible that membrane extensions bearing a high concentration of tetherin formed these apparent filaments. We observed these structures more consistently using the pre-embedding labeling technique than in cryosections, but failed to observe them at all in the absence of primary antibody or when pre-immune sera was employed. Thus we think they are specific structures worthy of additional investigation. Tetherin is intimately associated with the actin cytoskeleton [34], and focal areas of intense cortical actin were apparent underlying focal collections of tetherin in some sections (not shown). It is possible that actin or actin-associated proteins also contribute to the linear and perhaps helical filamentous connections we observed that were studded with tetherin.

In summary, tetherin was specifically associated with particle budding sites and was found between virions in chains. These data support a physical role for tetherin in retaining virion particles in restrictive cells.

## Materials and Methods

### Ethics statement

Animals for production of antisera were housed and handled at Cocalico Biologicals, Inc., Reamstown, PA. All animals were handled in strict accordance with good animal practice in accordance with NIH's Office of Laboratory Animal Welfare as reviewed by the Institutional Animal Care and Use Committee (IACUC) at Cocalico Biologicals (Animal Welfare Assurance number A3669-01).

### Cell lines and plasmids

293T cells (obtained from the American Type Culture Collection) were maintained in Dulbecco's Modified Eagle Medium (DMEM) supplemented with 10% fetal bovine serum (FBS) and penicillin/streptomycin (PS). A3.01 cells (a gift from Klaus Strebel, NIH) were propagated in RPMI-1640 supplemented with 10% FBS, 2mM L-glutamine, and PS. A HA-tetherin cell line stably expressing an N-terminal, HA-tagged tetherin was generated by retroviral transduction of 293T cells. Briefly, subconfluent 293T cells were infected with HA-tetherin encoding retroviral stocks overnight in 10 cm^2^ dishes in the presence of 8 µg/ml polybrene. The following day the cell monolayers were washed with PBS and incubated in fresh growth media. After 48 hours, infected 293T cells were diluted and propagated in growth media supplemented with 0.5 µg/ml of puromycin until single colonies were present. Single puromycin-resistant colonies were isolated and assayed for the stable expression of physiological levels of HA-tetherin by Western blotting.

The infectious HIV-1 molecular clone pNL4-3, and the *vpu*-deficient pNL4-3/Udel, have been described [15]. pHCMV-G is an expression plasmid encoding the vesicular stomatitis virus glycoprotein G (VSV-G) [35]. pCMV-HA.Tetherin is a plasmid encoding an N-terminal, HA-tagged tetherin obtained by PCR cloning of tetherin cDNA into the *Sal1-Xho1* sites of pCMV-HA (Clontech). The HA-Tetherin sequence was amplified by PCR cloning and inserted into the *Age1-Pac1* sites of the retroviral vector pQCXIP (Clontech) to generate pQCXIP-HA.tetherin.

### Retroviral stock production

HIV-1 viral stocks were generated by Fugene HD (Roche Diagnostics) co-transfection of 293T cells with the molecular clone, pNL4-3/Udel, and the vesicular stomatitis virus envelope glycoprotein expression plasmid pHCMV-G. Virus was harvested from transfected cell supernatants 48 hours post-transfection, filter-sterilized, and assayed for infectivity using TZM-bl indicator cells. Infectious titers were measured as β-galactosidase^+^ colony-forming units. A retroviral stock encoding HA-tetherin was prepared by co-transfecting 293T cells with pCL-Ampho [36], pQCXIP-HA-Tetherin, and pHCMV-G. Transfected cell supernatants were harvested 48 hours post-transfection, filter-sterilized, aliquoted, and stored at −80°C for future use.

### Generation of tetherin antisera

Anti-tetherin antiserum was elicited in rabbits at Cocalico Biologicals, Inc. (Reamstown, PA, USA) using a recombinant GST-tagged tetherin fusion protein. The tetherin fragment was composed of the entire ectodomain spanning amino acids 43–179 inserted into vector pGEX6p-1(GE LifeSciences). The tetherin fusion construct was concentrated from clarified bacterial sonicates using glutathione sepharose 4B beads. The GST-tag was excised with PreScission protease (GE Life Sciences) and the supernatants were then further purified using cation-exchange chromatography (HiTrap Q HP; GE Life Sciences). Rabbits were immunized with 250 µg of recombinant tetherin protein per dose until an endpoint antibody titer of 1.0×10^8^ was achieved. The rabbit anti-tetherin antiserum was assayed for reactivity and specificity in Western Blot and flow cytometry prior to final serum harvest.

### Flow cytometry

5×10^6^ 293T cells were propagated overnight in 10cm^2^ dishes; A3.01 cells were maintained in suspension culture. The cells were incubated for 24 h in the presence of 5000 U/ml IFN-α. On the following day the cell monolayer (293T) was washed twice with pre-warmed PBS and cells detached using 3 ml of Versene (0.2 g/l EDTA-4Na in PBS; Invitrogen) per dish. Cells were then pelleted and washed repeatedly with ice-cold PBS. Cells were resuspended in 2%BSA-PBS and allowed to incubate on ice for 10 min prior to the addition of primary antibody (rabbit α-tetherin) for 1 h on ice. The cells were washed twice with 2%BSA-PBS followed by the addition of Alexa Fluor 633-conjugated anti-rabbit IgG secondary antibody (Molecular Probes; Invitrogen) diluted to 1 µg/ml in 2%BSA-PBS. The mixture was allowed to incubate in the dark for 30 min on ice. The stained cells were analyzed using a FACS Canto (BD Biosciences) flow cytometer. Subsequent data analyses were performed using Flow Jo 7.2.4 (Tree Star).

### Cellular protease stripping experiments and sucrose density gradient analysis

In order to determine the presence of over-expressed tetherin on HIV-1 virions, 5×10^6^ HA-tetherin cells were plated per 10cm^2^ dish. The following day the cells were transfected with Fugene HD using 3 µg pNL4-3/Udel per dish. At 48 hours post-transfection, cells were washed twice with phosphate-buffered saline (PBS; pH 7.4). Cell monolayers were then incubated for 30 min at 37°C in 3.5 ml of Tris-Cl (pH 8.0), 150 mM NaCl, 5 mM CaCl_2_, and 5 µg/ml subtilisin (Sigma Aldrich). The digest was stopped by the addition of 3.5 ml of FBS-containing growth media plus protease inhibitors. Cellular supernatants were clarified by low-speed centrifugation and then purified by ultracentrifugation through a 20% sucrose cushion (100,000 *g* for 3 hours, 4°C). Pellets were then resuspended in 1 ml of PBS and overlaid onto linear 20–60% sucrose gradients. Ultracentrifugation of gradients was performed overnight at 100,000× *g* at 4°C. Equal 800 µl fractions were collected and density determined using a refractometer. Samples were diluted in PBS and concentrated using a microultracentrifuge (2 hours at 100,000 g; 4°C). Pellets were subsequently resuspended in 1× SDS-PAGE load buffer containing 100 mM DTT. Analysis of fractionated material was performed by Western blotting using anti-p24 hybridoma 183-H12-5C (obtained from Bruce Chesebro and Hardy Chen through the NIH AIDS Research Reference and Reagent Program) supernatants (1∶1000) and rabbit anti-tetherin polyclonal antisera (1∶2000).

Endogenous tetherin incorporation into HIV-1 virions was assessed by infecting A3.01 cells with VSV-G pseudotyped NL4.3/Udel. 1.0×10^7^ A3.01 cells were infected with VSV-G pseudotyped NL4.3/Udel at an MOI of 0.75. The cells were cultured for 3 days prior to the addition of 3000 U/ml IFN-α. On day 4 post-infection, the cells were pelleted at 350× *g* for 5 minutes at room temperature and washed twice with 10 ml PBS. The cells were resuspended in 5 ml serum-free growth media supplemented with 3 µg/ml TPCK-treated trypsin and incubated for 3 hours at 37°C. The protease activity of the supernatant was ablated by the addition of 5 ml of FBS-containing growth media plus protease inhibitors. The subsequent analysis of cellular digests was performed as described above.

### Electron microscopy

A3.01 cells were infected with VSV-G pseudotyped NL4.3/Udel and stimulated with IFN-α as described above. A subset of cell cultures were treated for 24 h prior to harvest with 5 µM indinavir to inhibit proteolytic particle maturation and assist in maintenance of virion morphology. At 48 hours, cells samples were fixed, sectioned, and stained for examination by transmission electron microscopy as previously described [37]. Pre-embedding immunogold labeling of samples for tetherin was performed by gentle fixation in 4% paraformaldehyde for 20 min at room temperature (RT). The cells were then extensively washed with 1% BSA-PBS and blocked with 1% gelatin-PBS for 30 min at RT. Primary antibody (rabbit anti-tetherin antisera) was diluted to 1∶300 in 1% gelatin-PBS and incubated with cells for 1.5 hours at RT with gentle agitation. The cells were extensively washed prior to the addition of secondary goat anti-rabbit IgG antibody conjugated to 6 nm (EM Sciences) gold beads for 1 hour at RT with gentle agitation. The cells were then washed extensively, fixed with 2.5% glutaraldehyde for 1 hr at RT, and postfixed with 1% osmium tetroxide at 4°C for 1hour. Dehydration with ethanol was performed and the cells were embedded in Eponate-12 resin. Regular 70 nm ultra-thin sections were produced, double stained with uranyl acetate and lead citrate, and observed under a Hitachi H7500 transmission electron microscope at 75 KV. Infected and control A3.01 cells were also analyzed by immuno-EM techniques on ultra-thin cryosections. Cryosections were generated by fixing cells in freshly made 4% paraformaldehyde in PBS for 20 minutes at RT. Tissue blocks were embedded in 10% gelatin. In a variation of this procedure, an additional fixation step with 0.7% gluteraldehyde for 40 minutes was performed. The cells were then washed extensively with PBS prior to infusion with 2.3 M sucrose, or alternatively 30% polypropylene glycol (PPG) as a cryoprotectant. The cell pellets were frozen using liquid nitrogen under controlled conditions (cryogen) and sectioned using a cryo-ultra microtome generating 70 nm cryosections. The sections were thawed on nickle grids with formvar supporting membranes prior to antibody labeling. The samples were blocked using 1% BSA-1% gelatin in PBS for 1 h at RT. Glycine was then used to block any free aldehyde groups present. The samples were incubated in 5 µg/ml of primary rabbit anti-tetherin antibody in 1% BSA/1% gelatin overnight at 4°C. The sections were washed extensively prior to the addition of secondary goat anti-rabbit IgG conjugated with 6 nm gold beads for 1 hour at RT. The sections were then air dried in a film of methylcellulose with 1% uranyl acetate prior to observation under a Hitachi H7500 transmission electron microscope. Control experiments included unlabeled cells, cells labeled in the absence of IFN stimulation, uninfected cells either treated or untreated with IFN, and samples from each experimental group in which the primary antibody was omitted during the immunolabeling procedure. A minimum of 25 cells were examined for each experimental arm and control when recording labeling positivity. A total of 70 cells and their associated viruses were examined to derive the mean number of immunogold particles present per virion (counting as positive those gold particles within 50nm of the virion) in the cryosection-immunolabeling experiments.

## Supporting Information

Figure S1 Micrographs from control experiments. All cells are A3.01 cells. (A) Untreated cells demonstrate lack of background surface labeling; secondary antibody only. Bar = 200nm. (B) Tetherin labeling in uninfected cells that were not treated with IFN. Rare collections of focal staining (arrow) were noted; quantitation included in Fig. S2. Bar = 200nm. (C) Tetherin labeling in IFN-stimulated, uninfected cells. Focal collections of immunostaining were much more common (see Fig. S2); representative focal staining shown. Bar = 100nm. (D) Infected cells, no IFN treatment, no primary antibody. No significant staining noted. Bar = 200nm. (E) Infected cells, no IFN treatment, primary and secondary antibody staining performed. Occasional focal staining noted (arrow). Bar = 200nm. (F) IFN-stimulated, infected cells, no primary antibody. No apparent immunolabeling observed. Bar = 200nm. (G) Indinavir-treated, infected cells, immunostaining in absence of primary antibody; only very rare gold beads noted. Bar = 200nm. (H) Cryosectioned cells stained without primary antibody; particles fail to label with secondary antibody conjugated to gold. Bar = 200nm. (I) Cryosectioned cells, intracellular vesicle with particles, no primary antibody used. A single gold particle noted away from virions (arrow). Bar = 200nm.
(8.79 MB TIF)

Figure S2 Quantitation of positive immunolabeling. (A) Quantitation of positive signal with pre-embedding labeling technique. 50 cells were counted for each experimental group. Shown is the percentage of cells labeled with 6nm gold particles. Normal bar represents unstimulated cells. Note that NL4.3/Udel (NLUdel)-infected cells following IFN stimulation demonstrated significant levels of specific staining. (B) Number of particle-associated gold beads in cryosection/immunolabeling experiments. 70 cells and all associated particles were counted in the case of the intact labeling protocol; 25 cells and associated particles were assessed for the control (no primary antibody) group. Shown is the mean ± SD number of gold particles overlying or within 50nm of virion particles. (C) IFN stimulation of A3.01 results in restriction of particle release. Shown are particles released from A3.01 cells infected with VSV-G-pseudotyped NL4.3 or NLUdel in the absence (−) or presence (+) of 3000 U/ml of IFN-α.
(0.80 MB TIF)

Figure S3 Additional controls. (A–C) NLUdel-infected A3.01 cells stimulated with IFN were subjected to cryosectioning and immunostaining as before, but with substitution of rabbit preimmune sera as the primary antibody. Isolated 6nm gold beads were observed in some fields, without apparent association with viral particles. Bars = 200nm. (D) NL4.3-infected and IFN-stimulated A3.01 cells only rarely demonstrated attached particles. Where present, the attached particles did demonstrate tetherin immunostaining on the outer surface, shown by pre-embedding labeling with anti-tetherin antisera and 6nm anti-rabbit immunogold. Bar = 100nm.
(4.04 MB TIF)
