# Supplementary figures and images for: Immunoelectron Microscopic Evidence for Tetherin/BST2 as the Physical Bridge between HIV-1 Virions and the Plasma Membrane
Source: PLoS Pathog. 2010 Feb 5;6(2):e1000749. doi: 10.1371/journal.ppat.1000749 (PMC2816691; doi:10.1371/journal.ppat.1000749)

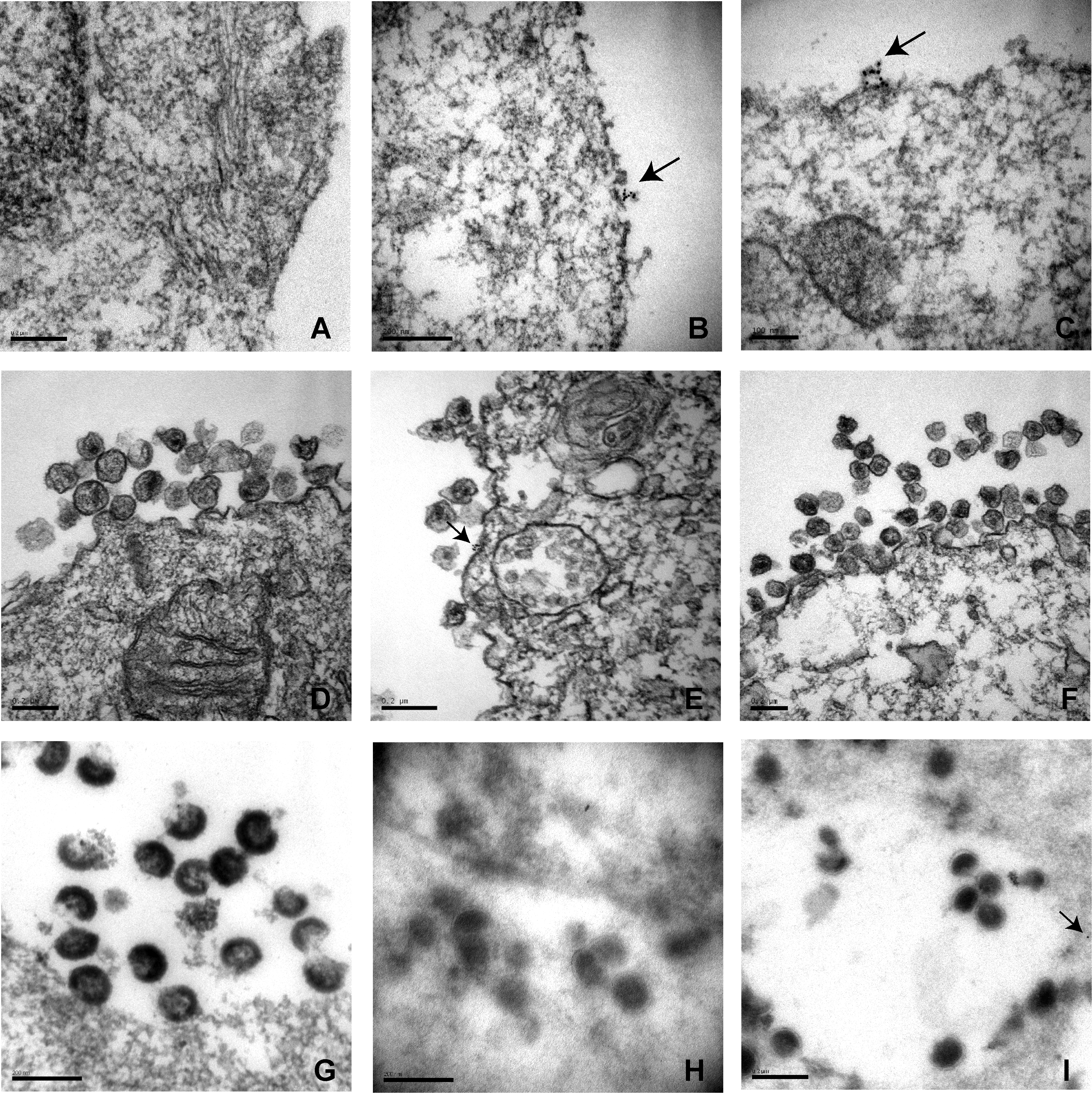

Supplement: Figure S1 — Micrographs from control experiments. All cells are A3.01 cells. (A) Untreated cells demonstrate lack of background surface labeling; secondary antibody only. Bar = 200nm. (B) Tetherin labeling in uninfected cells that were not treated with IFN. Rare collections of focal staining (arrow) were noted; quantitation included in Fig. S2. Bar = 200nm. (C) Tetherin labeling in IFN-stimulated, uninfected cells. Focal collections of immunostaining were much more common (see Fig. S2); representative focal staining shown. Bar = 100nm. (D) Infected cells, no IFN treatment, no primary antibody. No significant staining noted. Bar = 200nm. (E) Infected cells, no IFN treatment, primary and secondary antibody staining performed. Occasional focal staining noted (arrow). Bar = 200nm. (F) IFN-stimulated, infected cells, no primary antibody. No apparent immunolabeling observed. Bar = 200nm. (G) Indinavir-treated, infected cells, immunostaining in absence of primary antibody; only very rare gold beads noted. Bar = 200nm. (H) Cryosectioned cells stained without primary antibody; particles fail to label with secondary antibody conjugated to gold. Bar = 200nm. (I) Cryosectioned cells, intracellular vesicle with particles, no primary antibody used. A single gold particle noted away from virions (arrow). Bar = 200nm. (8.79 MB TIF) [file ppat.1000749.s001.tif]

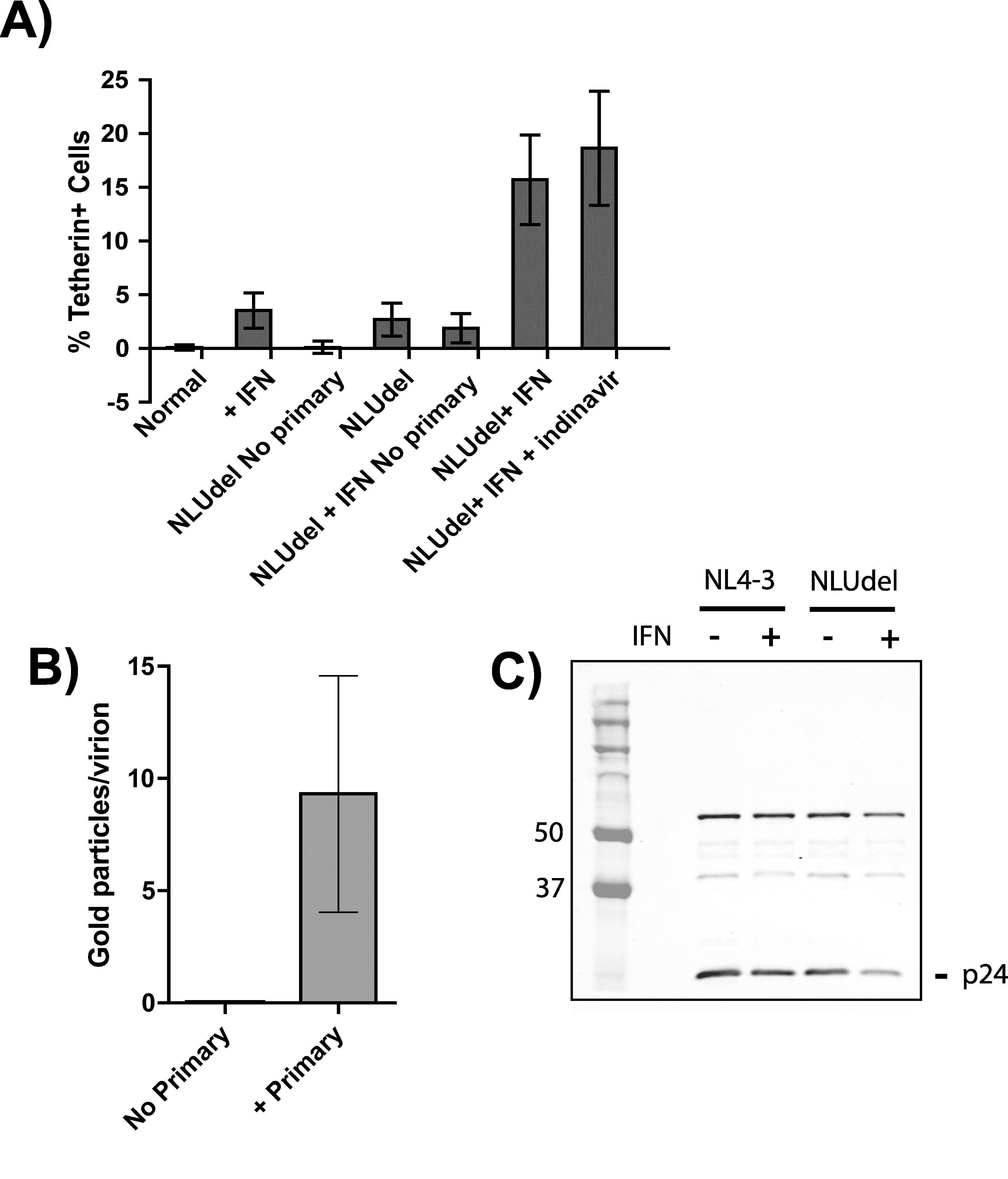

Supplement: Figure S2 — Quantitation of positive immunolabeling. (A) Quantitation of positive signal with pre-embedding labeling technique. 50 cells were counted for each experimental group. Shown is the percentage of cells labeled with 6nm gold particles. Normal bar represents unstimulated cells. Note that NL4.3/Udel (NLUdel)-infected cells following IFN stimulation demonstrated significant levels of specific staining. (B) Number of particle-associated gold beads in cryosection/immunolabeling experiments. 70 cells and all associated particles were counted in the case of the intact labeling protocol; 25 cells and associated particles were assessed for the control (no primary antibody) group. Shown is the mean ± SD number of gold particles overlying or within 50nm of virion particles. (C) IFN stimulation of A3.01 results in restriction of particle release. Shown are particles released from A3.01 cells infected with VSV-G-pseudotyped NL4.3 or NLUdel in the absence (−) or presence (+) of 3000 U/ml of IFN-α. (0.80 MB TIF) [file ppat.1000749.s002.tif]

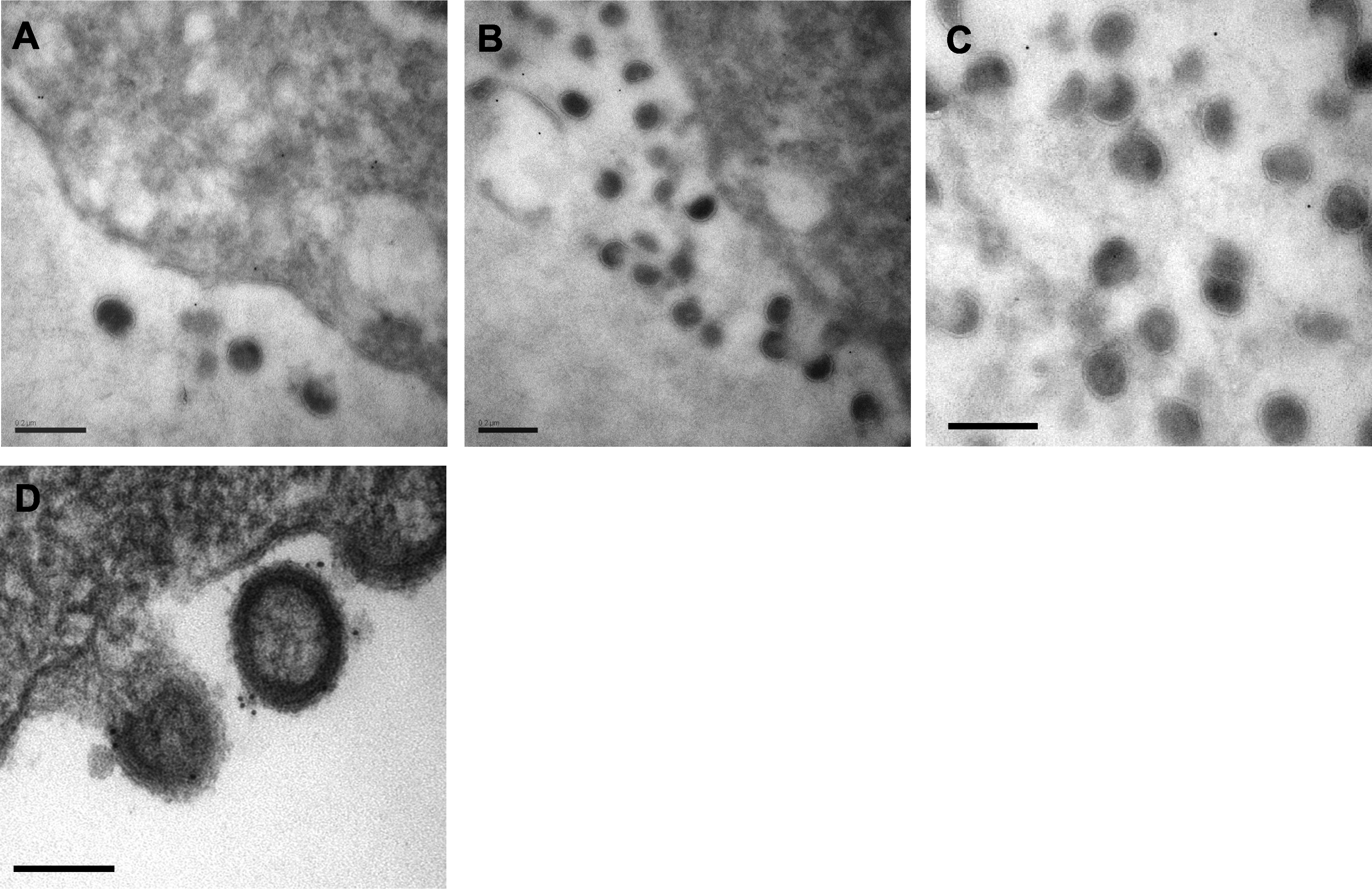

Supplement: Figure S3 — Additional controls. (A–C) NLUdel-infected A3.01 cells stimulated with IFN were subjected to cryosectioning and immunostaining as before, but with substitution of rabbit preimmune sera as the primary antibody. Isolated 6nm gold beads were observed in some fields, without apparent association with viral particles. Bars = 200nm. (D) NL4.3-infected and IFN-stimulated A3.01 cells only rarely demonstrated attached particles. Where present, the attached particles did demonstrate tetherin immunostaining on the outer surface, shown by pre-embedding labeling with anti-tetherin antisera and 6nm anti-rabbit immunogold. Bar = 100nm. (4.04 MB TIF) [file ppat.1000749.s003.tif]
